# Mpox in Africa: one year continental response outcomes and way forward

**DOI:** 10.1136/bmjgh-2025-023050

**Published:** 2026-09-15

**Authors:** Michel K Muteba, Kyeng Mercy, Yap Boum, Nebiyu Dereje, Merawi Aragaw Tegegne, Patrick Chanda Kabwe, Anthony Twyman, Yao S Atrah, Hieronyma Nelisiwe Gumede-Moeletsi, Charles Ugochukwu Ibeneme, Wazih Nji Cho, Laura Ambe, Beryl Njeba, John Masina, Joseph Armachie, Mamadou Saliou Kalifa Diallo, Sheillah Nsasiirwe, Polydor Mutombo, Denis Bunyoga, Deogratias Kakule, Olaniyi F Sanni, Rose Mary Nakame, Ibrahim Mamadu, Elizabeth Gonese, Viviane Kuissi, Priscilla Kusena, Linda Meta Mobula, Viviane N Fossouo, Usman Shehu, Selamawit Kebede, Bibi Kenta, Aurore Virayie, Emilio Hornsey, Cat Makisonbooth, Victor J Del Rio-Vilas, Natalie Fischer, Masresha Worku, Aarunima Bhatnagar, Eve Matthews, Abbie Harrison, Mosoka Papa Fallah, Gervais Folefack, Abou B Gaye, Mory Keita, Shanelle Hall, Otim Ramadan, Fiona Braka, Abdou Salam Gueye, Ngashi Ngongo, Jean Kaseya

**Affiliations:** 1 World Health Organisation Regional Office for Africa, Brazzaville, Congo; 2 Africa Centres for Disease Control and Prevention, Addis Ababa, Ethiopia; 3 UK Public Health Rapid Support Team (UK-PHRST), UK Health Security Agency, London, UK; 4 Emergency Preparedness and Response, World Health Organisation Regional Office for Africa, Nairobi, Kenya; 5 Immunisation, World Health Organisation Regional Office for Africa, Brazzaville, Congo; 6 National Centre for Naturopathic Medicine, Africa Centres for Disease Control and Prevention, Addis Ababa, Ethiopia; 7 International Federation of Red Cross and Red Crescent Societies, Geneva, Switzerland; 8 International Organisation for Migration, Geneva, Switzerland; 9 World Bank, Washington, District of Columbia, USA; 10 Food Programme, World Food Programme, Rome, Lazio, Italy; 11 United Kingdom Public Health Rapid Support Team, UK Health Security Agency, London, UK; 12 United Kingdom Public Health Rapid Support Team, UK Health Security Agency, London, England, UK; 13 Kingdom Public Health Rapid Support Team, London School of Hygiene & Tropical Medicine, London, UK; 14 United Nations Children’s Fund, New York, New York, USA; 15 Field Epidemiology Training Programme, UK Health Security Agency, London, UK; 16 Emergency Prepraedness and Response Programme, World Health Organisation Regional Office for Africa, Dakar, Senegal; 17 Institute of Global Health, University of Geneva Faculty of Medicine, Geneve, Switzerland; 18 Emergency Response, World Health Organisation Regional Office for Africa, Brazzaville, Congo

**Keywords:** Africa, Public Health, Health systems evaluation

## Abstract

**Background:**

Following the declaration of mpox as both a global and continental public health emergency in August 2024, the Joint Incident Management Support Team (IMST), co-led by the Africa Centres for Disease Control and Prevention (Africa CDC) and the WHO, was activated in Kinshasa, DRC to coordinate the continental mpox response. This article highlights the first year’s epidemiological and operational findings.

**Methods:**

Key performance indicators across eight IMST pillars (coordination, surveillance, laboratory, case management, infection prevention and control, risk communication and community engagement (RCCE), vaccination and research) were descriptively analysed using data from 28 African Union Member States from January 2024 to August 2025.

**Results:**

A total of 49 803 confirmed cases and 240 deaths (CFR 0.5%) were recorded across 28 countries. The response was supported with US$977 million as tracked by the IMST. Operational achievements included 87% surveillance completeness, 72% contact tracing completeness and improved average laboratory turnaround time from >5 days to 2 days. Laboratory testing capacity expanded from 2 to 27 sites in the DRC and from 1 to 56 decentralised sites in Burundi. RCCE activities reached 50 million people in 21 countries, with vaccine acceptance among high-risk groups rising from 44% to 87%. Operational research achievements included a responsive and functional research coordination mechanism, 24 (92%, 24/26) multi-country clinical and operational research studies implemented and 60% completion rate of the research data and material sharing framework for Africa. Only 938 000 doses (32%) of the 3.1 million vaccine doses delivered were administered.

**Conclusion:**

The joint continental IMST is a new benchmark for response governance, successfully mobilising resources and scaling up diagnostics under unified co-leadership. On the other hand, the evaluation revealed persistent structural challenges in last-mile vaccine delivery, contact tracing (due to stigma/funding) and weak regional collaborative research including fragmented regional research priorities. Future focus must shift from emergency action to durable, sustained investment in national and sub-national health security architecture using a multisectoral whole of government approach.

**WHAT IS ALREADY KNOWN ON THIS TOPIC**

- The African continent has faced the largest protracted mpox outbreak since 1970, though countries previously managed local outbreaks with minimal regional coordination.
- Stigma and social discrimination have historically served as major barriers to effective contact tracing and case reporting during epidemics like COVID-19, HIV and Ebola.
- The COVID-19 pandemic highlighted significant vulnerabilities in Africa’s access to medical countermeasures and vaccines, emphasising the need for equitable access and local manufacturing.

**WHAT THIS STUDY ADDS**

- It provides the first continent-wide analysis of the operational response under the Incident Management Support Team co-leadership of Africa CDC and WHO.
- The study documents a massive expansion of diagnostic capacity, such as Burundi scaling from 1 to 56 GeneXpert sites, reducing turnaround times from 72 hours to <4 hours which was one of the enablers in bending the curve.
- The findings reveal a “last-mile” delivery gap where, despite delivering 61% of projected vaccine doses, only 33% of those doses were administered by the end of Phase II.

**HOW THIS STUDY MIGHT AFFECT RESEARCH, PRACTICE OR POLICY**

- It advocates for African governments to honour the 15% Abuja Declaration pledge for health financing and to invest in local manufacturing of vaccines and other medical countermeasures.
- The results emphasise the need to integrate stigma reduction into clinical protocols and to recruit and train 2 million polyvalent community health workers by 2030 to strengthen surveillance and community engagement.
- The study highlights the necessity for a maximum 2 week ethical review for outbreak studies and the establishment of a regional data/material sharing framework to harmonise research priorities during emergencies.

## Introduction

 The African continent has been experiencing the largest protracted mpox outbreak since the first human case was detected in 1970.^1^ However, countries across Africa were largely left to manage local outbreaks with minimal regional or continental coordination, even when cross-border transmission was evident.^2^ In 2022, a global mpox outbreak occurred with an initial transmission of cases from Nigeria to Europe and by 23 July 2022, it was declared a public health emergency of international concern (PHEIC) with a total of 92 783 cases and 171 deaths globally.^3^ With the reduction of cases in Western countries and despite the increasing numbers in Africa, the PHEIC was lifted in 2023.^4^

By August 2024, amidst rising mpox case numbers and the expansion of clade Ib across multiple countries, the need for coordinated continental leadership to guide the response became critical. The Incident Management Support Team (IMST) was established which brought together 28 partner agencies under a unified “one plan, one team, one budget, one monitoring and evaluation framework” approach.^5^ The continental response was structured around 10 technical pillars: Surveillance and Health Information Management, Laboratory, Risk Communication and Community Engagement (RCCE), Vaccination, Case Management, Infection Prevention and Control (IPC), Logistics, Continuity of Essential Healthcare Services and Research.

This article presents the progress of key performance indicators (KPIs) achieved during the pre-declaration phase (January–August 2024) against the subsequent post-declaration phase I (September 2024–February 2025) and post-declaration phase II (March–August 2025).

## Methods

### Study design and setting

A retrospective descriptive analysis was conducted to evaluate epidemiological, operational and KPI performance data across eight response pillars where data were available (coordination, surveillance, laboratory, IPC, RCCE, Vaccination, research and case management). Continuity of essential services and logistics were not assessed. Assessment was limited to eight pillars because the logistics pillar was viewed as a facilitating pillar. Its essential commodity delivery function was already captured within the other technical pillar assessments. Furthermore, the Continuity of Essential Services pillar was a new introduction and obtaining the necessary milestone data for evaluation proved challenging, especially since this pillar was not yet integrated into existing country response systems. The study timeframe was from January 2024 to August 2025. The analysis included data from 28 African Union Member States that recorded confirmed cases of mpox during this period. Additional input was actively sourced from partners located within and external to the continent through the planning team.

### Data sources and collection

Surveillance, IPC and case management pillars gathered morbidity, mortality and tracing data via national IDSR systems, reports and reviews. Laboratory data PCR, sequencing, turnaround times relied on reference lab publications. Vaccination figures came from national immunisation systems and dashboards. RCCE used infodemic platforms, surveys and partner reports. Logistics tracked supply chains and customs records via country focal points, financial flows used a continental tracking mechanism and research drew from reports and workshops. Pre-declaration data were documented by reviewing scientific articles and consulting experts from Member States, Africa Centres for Disease Control and Prevention (Africa CDC), United Nations Children’s Fund (UNICEF) and the WHO.

Data was collected through a joint coordination mechanism in charge of planning within the continental IMST. The data was divided into three phases: pre-declaration, post-declaration phases I and II. The post-declaration phases represent the implementation period from September 2024 to August 2025 with the aims to bend the curve by intensifying response efforts, sustaining gains and building a legacy of the mpox response (online supplemental table 1).

### Data management and analysis

Surveillance data underwent automated and manual cleaning, deduplication and validation, while laboratory data were cross verified for consistency. Epidemic curves tracked weekly caseloads and Case Fatality Rates were calculated as deaths per confirmed case. Metrics included testing coverage (proportion of suspected cases tested), testing rate (samples tested vs received) and contact tracing completeness (contacts finishing 21-day follow-up). Laboratory turnaround time measured the interval from collection to result release. KPIs were summarised by response pillar against baselines, using descriptive statistics counts, percentages, medians and IQRs to characterise performance.

## Result

The results detail the operational activities executed post-IMST establishment (online supplemental table 1) and the performance of KPIs of eight response pillars, organised by the three distinct time periods under review (table 1).

**Table 1:** KPI performance across response pillars

Pillar	KPI	Target	Pre-declaration	During phase I September 2024–February 2025	During phase II March–August 2025	% of target met	Outcome
Coordination	Mobilise funds for the preparedness and response plans.	US$ 599.1 M	N/A*	$977 million	US$ 0	163%	Met target
Surveillance	Weekly reporting completeness (defined as proportion of countries reporting weekly).	≥80%	85%	89%	86%	100%	Met target
	Contact to case ratio.	20:1	1:1	3:1	3:1	15%	Did not meet target
	Contact tracing completeness.	≥70% (for 100% of countries)	N/A*	100% (n=3)	94% (for 9 reporting countries)	97% (for 11 countries)	Did not meet target
Laboratory	Testing rate (samples tested/samples arrived at the lab).	80%	<50%	95%	100%	121%	Met target
	Testing coverage (tested/suspected).	80%	<33%	55%	66%	75%	Did not meet target
	Median turnaround time ≤3 days.	≤3 days	>11days	≤3 days	≤2 days	100%	Met target
RCCE	People reached with key messages.	≥5 million	2 million	10 million (Cumulative)	50 million	600%	Met target
	% of category 1 Member States with established/institutionalised community feedback mechanism.	80%	75%	75%	91%	91%	Met target
	Self-reported vaccine acceptance.	80%	44%	87%	N/A*	108%	Met target
IPC	% reduction in healthcare-associated infections (HAIs) in targeted facilities among healthcare workers.	80% reduction	Varied by country (15–20% average prevalence in LMIC)	50%	83%	83%	Met target
Vaccination	Number of countries with regulatory approvals.	80% of active countries	0	26% (6)	74% (17)	94%	Met target
	Proportion of doses delivered of total projected target.	80%	0% (0)	13% (816 380)	48% (3 103 600)	61%	Did not meet target
	Proportion of doses administered of total doses delivered by country.	100%	0% (0)	13% (406 056)	33% (1 012 614)	33%	Did not meet target
Research	Functional and responsive research coordination mechanism.	Yes	None	Yes	Yes	100%	Met target
	Number of multi-country clinical and operational research implemented.	Complete 20 of 26 studies	N/A*	17	24	24	Met target
	Completion rate of the research data and material sharing framework for Africa.	70%	N/A*	15%	70%	60%	Did not meet target
Case management	Case fatality ratio.	<1%	0.57%	0.57%	0.47%		Met target

IPC, Infection Prevention and Control; KPI, key performance indicator; LMIC, low and middle income countries; N/A, not applicable; N/A*, data not available; RCCE, risk communication and community engagement.

### Pre-declaration phase (January 2024–August 2024)

**Figure 1:**
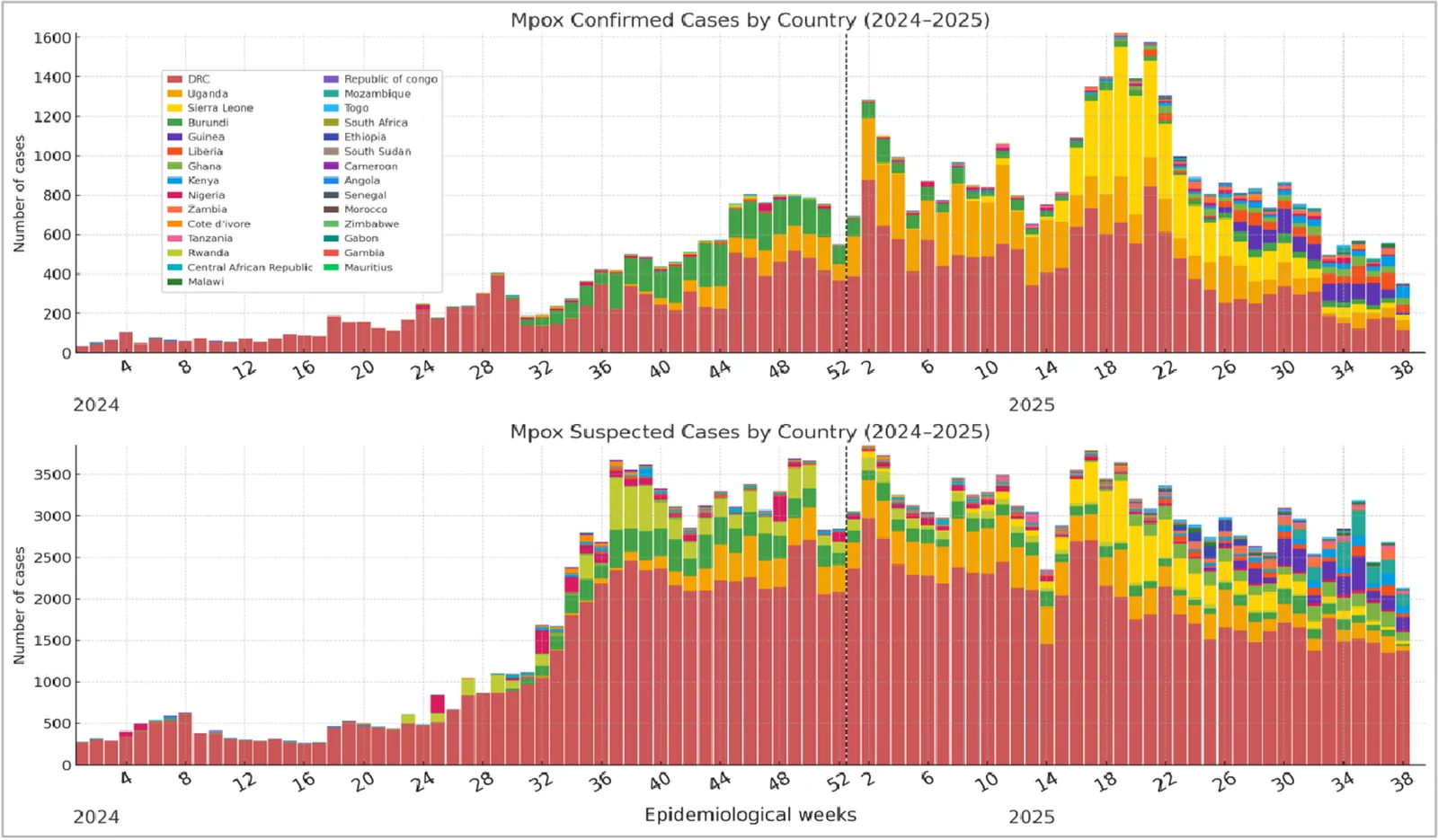
Epi curve of confirmed and suspected cases by country in Africa.

**Figure 2:**
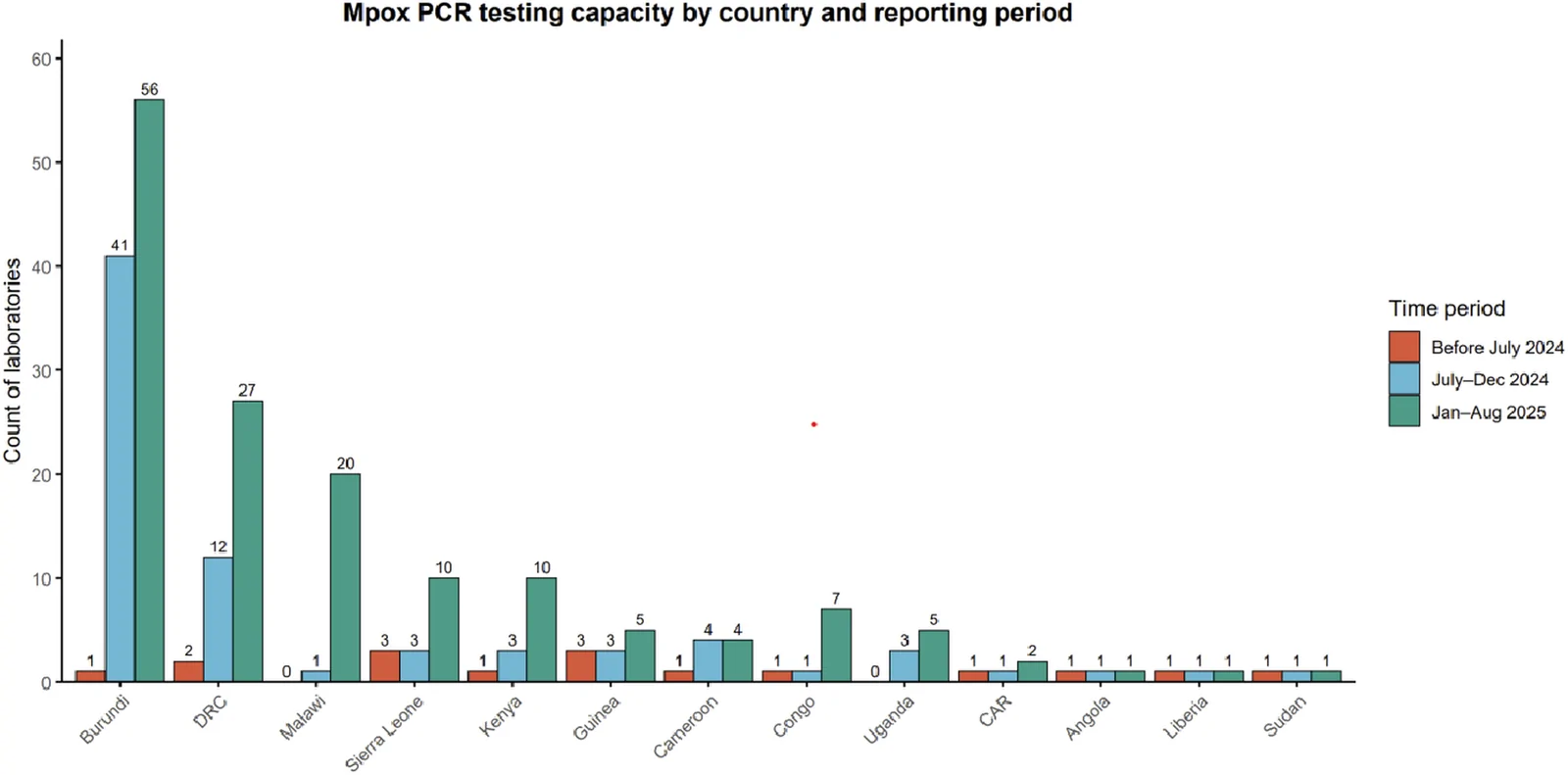
Mpox testing laboratories decentralisation evolution in Africa.

During the pre-declaration phase, 12 countries (7 reported clade 1b) had reported a total of 16 620 cases and 577 deaths among suspected cases and 4175 confirmed cases with 24 deaths (CFR: 0.57%). An increasing trend in cases was observed during this phase, with an average weekly confirmed case and suspected case of 134 and 536, respectively (figure 1). During this period, surveillance was mainly passive and contact tracing was not performed. On average, one laboratory per country was able to perform Mpox diagnosis, with testing largely centralised in the affected countries and <33% of all suspected cases were tested. High-burden countries like DRC and Burundi had 2 and 1 laboratories respectively with capacity to perform GeneXpert-based testing (figure 2). The laboratory turnaround time ranged from a minimum of 3 days to a maximum of 90 days, with an estimated average of approximately 11 days. During this phase, there was no continental financial tracking mechanism (FTM) in place and partner support to countries was not coordinated at the continental level. There were no vaccines on the entire continent and only two countries had approval from their national regulatory authorities. RCCE, IPC and case management were mainly locally coordinated with no standardisation or national guidance readily available. Tecovirimat was the primary medication available for treatment. Prior to the outbreak declaration, the research ecosystem was fragmented, marked by the lack of a functional continental research coordination mechanism, standardised guidance for outbreak research and formal frameworks for regional data/material sharing and integrated community-centred protocols.

### Post-declaration phase I (Response plan v.1: September 2024 to February 2025)

The initial continental response plan was costed at US$499 million, and donor pledges reached over US$1.1 billion, of which the FTM monitored US$977 million (88.8%). During this phase, the continent observed a steady rise in cases and outbreaks in 12 new countries (six new countries with Clade Ib). The average weekly confirmed case count was 651, with a CFR of 0.47% (95% CI 0.16% to 1.35%).

Laboratory testing sites were strategically expanded in high-burden countries, such as the DRC (from 2 to 12 laboratories) and Burundi (from one central laboratory to 41 decentralised sites), ensuring accessibility, adequate technical capacity, trained personnel, reliable GeneXpert systems, continuous energy supply, and availability of reagents and consumables. The continental testing coverage also increased from <33% (95% CI 32.7% to 33.3%) to 55% (95% CI 54.7% to 55.3%) of all suspected cases tested. A total of 309 586 laboratory test kits were delivered to 38 countries. On disease prevention communication, a total of 10 million persons were reached with key messages on mpox, and by the end of Phase I, vaccine acceptance had increased from 44% to 87% and 816 380 vaccine doses were delivered to eight countries, 406 056 (50%) of which were administered across five countries. This phase saw the establishment of a functional continental mpox research coordination mechanism, including a research and development blueprint jointly developed by Africa CDC, WHO and other partners. This facilitated the development and implementation of a multi-country social and behavioural study in nine countries, a regional data/material sharing framework, outbreak research guidance, community-centred protocols and capacity building for local researchers.

### Post-declaration phase II (Response plan v.2: March 2025 to August 2025)

Phase II continued the strategic response with a budget of US$429.6 million, though a US$224 million funding gap remained. Despite the initial case count increase in Phase II (average weekly confirmed cases of 937 and a CFR of 0.59%), the epidemiological trend showed a subsequent steady decline by the end of the phase (figure 1), with only three new countries reporting outbreaks. The strategic focus shifted to massive decentralisation of laboratory testing capacity and increased vaccine delivery. Testing capacity expanded significantly, with the DRC reaching 27 sites, Burundi establishing 56 GeneXpert sites and Sierra Leone: from 3 to 10 laboratories (figure 2); overall continental testing coverage increased from 55% to 66%, and the median turnaround time dropped from 3 days to 2 days.

The number of countries with vaccine regulatory authorisation nearly tripled (from 6 to 17), and a total of 2.28 million additional doses were delivered, 606 558 of which were administered. DRC accounted for 70% of the total vaccinated population (table 2). Seventeen multi-country clinical and operational mpox research studies were implemented by African scientists to assess the effectiveness of intervention in the African context and included social behavioural aspects, epidemiology, diagnostic tools, therapeutic, adaptive clinical trial platforms for the treatment of mpox (MOSA study) and vaccines.

**Table 2:** Mpox doses received and administered during phase I and II in Africa

Country	MVA-BN doses received	LC16 doses received	Total doses received	1st dose administered	2nd dose administered	Total doses administered
DRC	929 660	1 550 000	2 479 660	603 338	52 955	695 387
Rwanda	45 520	–	45 520	15 158	5573	20 721
Nigeria	39 100	–	39 100	23 255	3726	26 981
Uganda	209 060	–	209 060	109 900	–	109 900
CAR	12 300	–	12 300	592	–	592
Sierra Leone	206 300	–	206 300	149 504	–	149 504
Liberia	10 800	–	10 800	4509	2680	7189
South Africa	10 700	–	10 700	1111	–	1111
Cote d’Ivoire	11 300	–	11 300	929	–	929
Kenya	10 700	–	10 700	–	–	
Angola	67 000	–	67 000	300	–	300
Zambia	1160		1160	–	–	
Total	1 553 600	1 550 000	3 103 600	908 596	64 934	1 012 614

To ensure standardised care across a pool of over 4700 clinicians, six mpox clinical job aids and adapted global treatment guidelines were developed and disseminated. Furthermore, logistics pipeline was established: essential medical commodities sufficient to treat over 10 000 patients were distributed to affected AU Member States, and nutritional support was provided to more than 2000 patients in treatment centres. There was no effective therapeutic for treatment of mpox following the results of the PALM007 clinical trials that found tecovirimat ineffective against clade 1b disease.

Implementation of IPC strategies resulted in achieving 83% reduction in healthcare workers (HCWs) infections across targeted facilities, against a 100% reduction target in DRC, Burundi and Uganda. DRC reported a reduction in HCW infections from 104 to 52, Burundi reduced HCW infections from 4 to 0 and Uganda recorded no HCW cases during the reporting period. An early continental IPC/water, sanitation and hygiene (WASH) needs assessment highlighted persistent gaps, including insufficient trained personnel, inadequate isolation capacity, weak WASH infrastructure, particularly in schools, and recurrent stock-outs of essential IPC supplies. Interventions to improve these gaps led to over 500 health workers in outbreak epicentres receiving hands-on IPC training with demonstrated improvements in pre-post assessment scores. A major milestone included the development and novel intervention of the IPC and WASH measures for Home-based Care and Isolation for Mpox in Resource-Limited Settings operational guide, developed in response to the overwhelming case loads.

## Discussion

This study provides the first continent-wide analysis of the operational response pillars of Africa’s mpox outbreak under the continental IMST coordination structure. The findings reveal a positive shift in all indicators post the establishment of the IMST compared with the pre-declaration phase, demonstrating the unique strengths brought by the IMST to the response. Crucially, the declaration of mpox as a PHECS and PHEIC may have catalysed substantial progress: 14 of the 20 assessed KPIs ultimately met their targets. This success was, however, tempered by persistent critical gaps observed in contact tracing, vaccine delivery and last-mile logistics, including sample collection and transportation. One very remarkable outcome following the establishment of the IMST was the development of a costed continental preparedness and response plan under the “4 ONEs” principle (One Team, One Plan, One Budget, One monitoring and evaluation Framework). Through this coordination mechanism, at least 28 partners, over US$1.1 billion and six million doses of vaccine were mobilised, amidst the reduction of official development assistance and freeze of the United States Government funds. Similar coordination mechanisms like the Taskforce for Novel Coronavirus (AFTCOR) established early in the pandemic, which led to the launching of Africa Joint Continental Strategy for COVID-19, and establishment of the Africa Medical Supplies Platform for procurement, also demonstrated commendable impact in improving Africa’s access to vaccines and medical countermeasures.^6^ Nonetheless, the IMST was historic in its design, bringing Africa CDC and WHO as co-leads, using a single response plan, budget and monitoring and evaluation. The IMST model set an exemplary mechanism that could be used during response to future outbreaks in Africa.

Prior to the establishment of the IMST (pre-declaration phase), low weekly caseloads were attributed to both weak surveillance capacity and sub-optimal laboratory capacities to test in Africa.^4^ In addition, the newly introduced clade Ib, which was spreading in sexual networks, was introduced in only seven countries at the time.^7^ By contrast, the number of affected countries rapidly increased to 20 in phase I and 25 in phase II, driven largely by clade 1b outbreaks in the DRC and Eastern Africa due to cross border. Travellers, informal traders and truck drivers were among key populations driving cross borders spread due to high mobility a similar pattern observed during the acute phase of the HIV/AIDS pandemic.^8 9^ The expanding outbreak was reflected by a surge in weekly confirmed cases, which increased from 651 in Phase I to 937 in Phase II. The increase in case counts was largely attributable to increased viral spread within sexual networks, households and communities across several affected countries, enhanced surveillance and testing capacity.^9^

A critical operational weakness was observed in contact tracing, evidenced by the low contact-to-case ratio (<5 contacts listed per case), indicating a significant breakdown in the identification and follow-up necessary to interrupt transmission chains.^10^ This has largely been attributed to the weak health infrastructure, stigma (hindering disclosure of sexual contacts) and limited funding.^11^ Such failures have been previously documented as major barriers in epidemics like COVID-19, HIV and Ebola, primarily driven by underlying stigma and social discrimination that challenged prompt reporting and contact disclosure. This pattern also resembles the 2022 multinational clade IIb outbreak, which demonstrated the virus’s capacity for sustained transmission in sexual and household contact networks.^12 13^ Furthermore, countries faced a substantial financial burden related to the recruitment, training and deployment of community health workers (CHWs), which directly hampered optimal surveillance, community IPC efforts and contact tracing.

Established health facilities achieved the <3% target with a CFR of 2.1% among hospitalised confirmed cases; conversely, in studies conducted in remote communities, CFRs were significantly higher driven by delayed care seeking, poor treatment access and the complexities of managing co-morbidities such as HIV co-infection.^14^ In Clinical Management, continental “trainer-of-trainers” workshops created a strong cadre of clinicians, supported by tailored mpox clinical job aids and updated global treatment guidelines to ensure high-quality care. Early mobilisation of a pool of trained frontline HCWs is critical to ensure quality of care during epidemics and this approach was demonstrated during previous viral epidemics.^15 16^ A robust logistics system ensured uninterrupted delivery of essential medical supplies and nutritional support, strengthening patient care capacity. Learning from the COVID-19 pandemic, that exposed vulnerabilities in access to medical countermeasures, early investments in prepositioning of medical supplies is critical to ensure patients received the highest quality of care.^17^

IPC interventions such as HCWs capacity building, IPC kits delivery and health facility mentorship improved IPC compliance and supported reductions in HAIs among HCW infections, especially DRC, Burundi and Uganda as these were priority countries with the highest cases being reported during this study period. Adequate training, IPC supplies and technical assistance has shown to improve behavioural change to prevent breach in IPC and thus minimise risk of exposure to infectious diseases in healthcare settings.^18 19^

Vaccine administration was low partly due to delays in delivery and in the disbursement of operational funds. By Phase II, the proportion of doses administered of total doses delivered by country only reached 33%, indicating significant challenges in vaccine rollout and logistics, even though the proportion of doses delivered of total projected target doses reached 61%. This shortfall in vaccine delivery further revealed the gaps in access to vaccines and medical countermeasures highlighted during the 2022 mpox multicounty outbreak and the COVID-19 pandemic. However, self-reported vaccine acceptance in high-risk groups improved dramatically from 44% during pre-declaration to 87% in Phase I, demonstrating the contributing gains made in RCCE efforts. This high acceptance was due to wide community engagement leveraging social media. Social media mobilisation strategies demonstrated effective during Ebola outbreaks^20^ were adapted, including the use of existing community structures to increase trust in responders, while social listening tools pioneered during COVID-19^21 22^ enabled real-time message adjustment. The use of existing CHWs in DRC, that were subsequently trained in Community based Surveillance in South Kivu, allowed for improved detection of cases.

The official declaration may have contributed to a rapid scale-up of continental laboratory testing capacity. A demonstrable increase in testing coverage from <33% before the official declaration to 66% during Phase II was observed. This improvement may have been improved by the strategic expansion of diagnostic capacity in high-burden countries. For example, DRC increased its testing sites from 2 to 27 within 1 year. Similarly, Burundi successfully scaled up its laboratory network to include 56 GeneXpert sites, drastically reducing the turnaround time for test results from 72 hours to <4 hours. The significant reduction in the diagnostic turnaround time achieved by Burundi has been cited as a major operational best practice. This rapid processing is considered a key determinant in improving the timeliness of case detection and ensuring prompt patient isolation, which is essential for breaking active transmission chains.^23 24^

The establishment of a functional research coordination mechanism created an avenue for the engagement of National Public Health Institutes and Ministries of Health for knowledge sharing and peer-to-peer support. This mechanism supported the implementation of over 24 multi-country clinical and operational studies led by African scientists, covering social-behavioural, epidemiological, therapeutic, vaccine efficacy and diagnostics. Integration of research to response efforts would strengthen the overall response by providing the evidence needed and bridging the know-to-do gap. However, this research effort must be supported by expedited local research ethical clearance, dedicated funding and financial/logistical support for studies and collaboration across all mpox response pillars. Moreover, sustained investment is required to strengthen regional data sharing, harmonise research priorities via a One Health approach and establish dedicated mechanisms for consistently analysing operational research challenges.

Operational support provided by the IMST was critical for sustaining the multi-country mpox response from January 2024 to August 2025, despite significant logistical hurdles. The IMST centrally managed the supply chain, delivering 309 586 laboratory test kits (valued at US$10.9 million) to 38 countries. This support was essential for equipping and sustaining response efforts in hard-to-reach zones. These gains mirror the rapid laboratory scale-up during COVID-19, when GeneXpert and mobile PCR platforms were leveraged across Africa,^25 26^ and echo the role of decentralised testing during Ebola outbreaks.^27 28^ However, in countries grappling with complex humanitarian crises, such as DRC, logistical hurdles remained formidable. Sample collection and transportation challenges persisted, particularly in the Eastern regions, where insecurity and limited access rendered routine operations nearly impossible. In addition, bottlenecks in cross-border logistics and customs clearance highlighted structural weaknesses absent in more globally financed responses such as COVID-19, reinforcing the need for a multisectoral, whole-of-government approach in designing response systems in Africa.

### Study limitation

The analysis was limited to 8 of 10 response pillars due to unavailable data for Logistics and Continuity of Essential Services. Data completeness varied, particularly for contact tracing, IPC and research metrics. Furthermore, reliance on self-reported data (surveys) could have potential reporting bias. Another limitation is the inability to directly attribute all the successes registered to the declarations as some gains could be attributed to routine programme implementations which coincided with the period of declaration. However, the mixed-method approach facilitated the acquisition of additional qualitative data from partners and Member States to complement the data analysed.

## Conclusion

The coordinated continental response to the mpox outbreak, spearheaded by the historic co-leadership of the Africa CDC and WHO under the “4 ONEs” principle, established a new benchmark for epidemic governance in Africa. While demonstrating commendable success in securing political will, mobilising significant resources, rapidly scaling up laboratory diagnostics and achieving high vaccine acceptance through coordinated RCCE, the response simultaneously exposed persistent structural weaknesses. The key challenges and bottlenecks were seen in last-mile vaccine delivery, the critical breakdown of contact tracing driven by underlying stigma and financial hurdles for CHWs and the persistent inequity in clinical outcomes between established centres and remote regions, and insufficient national capacity to sustain IPC/WASH functions despite the strong regional progress observed. Several countries continued to face chronic stock-outs of essential IPC supplies, limited permanent isolation capacity and inadequate WASH infrastructure, especially in schools and peripheral health facilities. These constraints highlighted the need for durable system-level investments beyond emergency deployments. Notwithstanding these challenges, the achievement of the 83% reduction in HCW infections across targeted facilities against an 80% reduction target demonstrates the value of structured continental coordination, harmonised guidance and focused capacity building. Moving forward, the focus must shift from emergency response to sustained investment in the national and sub-national health security architecture using a multisectoral approach.

### Way forward

The continent-wide analysis underscores the need to shift from pure emergency response to sustained investment in national health security, leveraging the “4 ONEs” governance model. Key recommendations focus on four areas:

1. African governments must urgently honour the 15% Abuja Declaration pledge for sustained financing of essential functions (surveillance, CHW deployment) and system resilience. Strategically invest in local manufacturing of vaccines and diagnostics to meet the 60% local production goal by 2040, overcoming import dependence. Strengthen regional knowledge platforms to publish interim observations and advocate for a maximum 2 week ethical review for outbreak studies.
2. Establish dedicated, sustained funding for the recruitment, training and remuneration of 2 million polyvalent CHWs by 2030 for robust surveillance and IPC. Mandate the integration of stigma reduction into RCCE and clinical protocols to foster trust and improve contact tracing effectiveness.
3. Invest in national/regional cold chain and logistics to close the gap between vaccine doses delivered and administered, developing tailored last-mile strategies for high-risk groups. Prioritise the decentralisation of clinical services and essential commodities to remote regions to reduce care-seeking delays and narrow the persistent CFR gap.
4. Sustain financing for decentralised laboratory networks (eg, GeneXpert) and rapid turnaround practices for timely detection. Mandate multi-sectoral approaches to fix cross-border logistics and customs to prevent supply bottlenecks and engage humanitarian agencies to facilitate access and secure countermeasures in conflict zones.

## Supplementary material

10.1136/bmjgh-2025-023050 online supplemental file 1

10.1136/bmjgh-2025-023050 online supplemental table 1

## Data Availability

All data relevant to the study are included in the article or uploaded as supplementary information.
