# Supplementary material for: Mpox in Africa: one year continental response outcomes and way forward
Source: BMJ Glob Health. 2026 Sep 15;11(9):e023050. doi: 10.1136/bmjgh-2025-023050 (PMC13583846; doi:10.1136/bmjgh-2025-023050)
Supplement: Supplementary file 1 [file bmjgh-11-9-s001.pdf]

BMJ Global Health Author Reflexivity Statement

Adapted from Morton, B., Vercueil, A., Masekela, R., Heinz, E., Reimer, L., Saleh, S., Kalinga, C., Seekles, M., Biccard, B., Chakaya, J., Abimbola, S., Obasi, A. and Oriyo, N. (2022), Consensus statement on measures to promote equitable authorship in the publication of research from international partnerships. Anaesthesia, 77: 264-276. https://doi.org/10.1111/anae.15597

**Study conceptualisation**	
1. How does this study address local research and policy priorities?	The study advocates for African governments to honor the 15% Abuja Declaration pledge for health financing.<br><br>The research addresses the need for a regional data and material sharing framework to harmonize continental research priorities<br><br>It also highlights the priority of recruiting and training 2 million polyvalent Community Health Workers by 2030 to strengthen local surveillance
2. How were local researchers involved in study design?	The analysis was conducted under the IMST co-led by Africa CDC and WHO, involving global and regional experts from partner organisation involved in the response
**Research management**	
3. How has funding been used to support the local research team(s)?	The manuscript states that the authors received no specific funding for this work.
**Data acquisition and analysis**	
4. How are research staff who conducted data collection acknowledged?	Data was consolidated through the planning team within the IMST and all contributing individuals from the Africa CDC, WHO, and various international partner agencies are listed as contributing authors.
5. How have members of the research partnership been provided with access to study data?	A shared folder was created by the planning team where all the data from the various response pillars were uploaded. All partners supporting the response were granted access to the folder.<br>All data related to this manuscript has been included in this article which will be available to all regardless.
6. How were data used to develop analytical skills within the partnership?	Partners like the UKHSA were able to use the data to provide capacity building on advance analytics to the data team within the IMST.
**Data interpretation**	
7. How have research partners collaborated in interpreting study data?	Interpretation occurred through the Joint IMST which held weekly coordination meetings with over 28 partners.<br><br>A continental intra-action review was conducted to collectively interpret response outcomes and identify structural gaps
**Drafting and revising for intellectual content**	

8. How were research partners supported to develop writing skills?	A core writing team were assigned to develop the first draft which was then shared to the broader team for inputs. This was one of the ways capacity was built within the partnership framework.
9. How will research products be shared to address local needs?	Research findings are disseminated through continental guidelines, clinical job aids, and updated treatment guidelines provided to frontline clinicians.
**Authorship**	
10. How is the leadership, contribution and ownership of this work by LMIC researchers recognised within the authorship?	The leadership is reflected in the joint senior authorship held by representatives from the Africa CDC and WHO Africa region.<br>The corresponding author is also from LMIC
11. How have early career researchers across the partnership been included within the authorship team?	While professional titles are not listed, the authorship includes a wide range of contributors including early career researchers like Wazih Cho
12. How has gender balance been addressed within the authorship?	The manuscript lists a diverse group of male and female contributors across the various partner organizations. The corresponding author is a female and the first author is a mix of both genders.
**Training**	
13. How has the project contributed to training of LMIC researchers?	Through the IMST, early career researchers in countries have been supported by continental research experts in drafting country specific mpox manuscripts.
**Infrastructure**	
14. How has the project contributed to improvements in local infrastructure?	It significantly expanded laboratory networks, such as increasing sites in the DRC from 2 to 27 and in Burundi from 1 to 56
**Governance**	
15. What safeguarding procedures were used to protect local study participants and researchers?	Data were analyzed in aggregate without personal identifiers to protect participant privacy
