# Supplementary material for: Mpox in Africa: one year continental response outcomes and way forward
Source: BMJ Glob Health. 2026 Sep 15;11(9):e023050. doi: 10.1136/bmjgh-2025-023050 (PMC13583846; doi:10.1136/bmjgh-2025-023050)
Supplement: Supplementary file 2 [file bmjgh-11-9-s002.docx]

**Supplementary Table 1: Key interventions implemented during the post-declaration phases (September 2024 – August 2025**

| **Response pillars** | **Phase I key interventions** | **Phase II key interventions** |
| --- | --- | --- |
| Coordination | Developed and implemented the Mpox Preparedness and Response Plan with a $599 million budget.  Operationalized the IMST with weekly coordination meetings, including bilateral sessions with 25 partners and affected countries.  Developed and cost continental preparedness and response plan for phase I.  Launched Financial Tracking Mechanism (FTM) to ensure transparency in resource allocation and use.  Conducted a continental intra-action review (IAR) and supported Burundi, Uganda and Central African Republic with intra-action reviews. | Developed Mpox Response and Legacy Plan 2.0.  Provide technical and financial support to affected countries for effective country level response.  Mobilized 28 partners under the IMST coordination.  Developed costed plan for phase II.  Joint Mission to Sierra Leone, Togo, Malawi and Uganda.  Organized 16 partner coordination meetings, 16 country coordination meetings, and 50 IMST coordination meetings, enabling active participation and contribution from all stakeholders involved in the response. |
| Surveillance | Established a joint surveillance database between Africa CDC and WHO to improve analysis.  Developed and published standardized mpox surveillance protocols validated by 20 AU Member States.  Developed and supported national EBS systems in South Africa, Rwanda, and Burundi.  Convened eight countries to coordinate cross-border surveillance.  Trained 70 epidemiologists and 38 country surveillance focal points on harmonized indicators and systems. | Deployed more than 500+ community health workers in case identification and contact tracing.  Trained over 10,000 community health care workers (CHWs) and active engagement of CBOs in surveillance across multiple countries.  Maintained harmonized joint database for continental response.  Organised webinars for bringing together 850 PoE surveillance officers on early detection.  Technical support was provided to Sierra Leone, Mozambique, and Togo, standardizing the reporting of key surveillance indicators. |
| Laboratory | Expanded lab network in DRC (from 2 to 12 labs) and Burundi (from 1 to 41).  Delivered $5.74 million in laboratory infrastructure and consumables including over 26,700 test kits and reagents to 18 countries.  Trained 167 lab staff and held a regional training of trainers (ToT) for sequencing and ISO standards.  Developed continental Mpox laboratory testing strategy.  Supported data harmonization and partner coordination through regular DRC Lab Pillar meetings. | Expanded lab network in DRC (from 12 to 27 labs) and Burundi (from 41 to 56).  Supported the development and dissemination of Mpox laboratory decentralization plan in Burundi.  Developed and launched continental guideline for laboratory testing capacity decentralization.  Trained 248 lab officers on testing, biosafety, genomic and bioinformatics, sample management, GeneXpert use, and qPCR operations. |
| Vaccination | Over 816,000 mpox vaccine doses were delivered and 343,801 individuals vaccinated across 8 countries.  Constituted a continental Mpox vaccine allocation and distribution mechanism via the Access and allocation independent technical review committee (TRC).  Conducted planning workshop with 13 countries and supported 17 countries in reviewing their vaccination plans.  Developed an mpox vaccination toolkit and hosted webinars on regulatory pathways and implementation.  Supported rollout in 5 countries with tailored strategies for high-risk groups.  Initiated engagements with Bavarian Nordic and Coalition for Epidemic Preparedness Innovations (CEPI) to advance Africa-based vaccine manufacturing.  Supported regulatory processes including Emergency Use Authorizations (EUA) and National Immunization Technical Advisory Group (NITAG), approvals in 13 countries and facilitating age-specific vaccine authorizations to accelerate access. | Delivered more than 180, 000 dose of vaccines to mpox affected countries.  Mobilized additional donation and procurement of mpox vaccine resources to expand global and continental residual stock.  Conducted stock taking and experience sharing converge with 17 countries.  Expanded mpox vaccine rollout in 3 additional countries.  Provided technical assistance and operational support to scale up mpox vaccination rollout.  Supported regulatory processes including Emergency Use Authorizations (EUA) and NITAG approvals in 5 additional countries and facilitating age-specific vaccine authorizations to accelerate access. |
| RCCE | Developed RCCE plans for social events and CHW guides to enhance interpersonal communication.  Supported infodemic surveillance using tools like U-Report and call centers in Kenya and DRC.  Launched a campaign that reached 13.5 million people via short message service (SMS), social media, and home visits.  Conducted community rapid assessments (CRA) in six countries to evaluate knowledge, attitudes, and behaviors.  Developed rapid qualitative assessment protocols with Kenya, Burundi, and Uganda. | Trained more than 1100 community health workers and produced guidelines, and national response plans.  Developed RCCE messages using human centered design approach.  RCCE frameworks and improved community feedback mechanisms including infodemic management.  Conducted three rounds of CRA to understand behavioural trajectories across population groups.  Provided technical support to DRC, Malawi, Kenya, Nigeria, Ghana, and Togo through the provision of technical documents, updated messages, and operational guides on RCCE. |
| Research | Coordinated 48 research projects tracked via CEPI dashboard.  Co-developed protocols on social behaviour, vaccine uptake, and therapeutic trials.  Supported implementation in DRC, Burundi, Rwanda, Uganda, and CAR.  Enabled multi-country phase III and immunogenicity trials with African PIs and institutions.  Established WHO ethics approval and institutional IRB clearance for several studies. | Developed and disseminated research and ethical guidelines.  Built capacity of local researchers through targeted training and mentorship.  Preliminary results from multi-country mpox social behavioral studies. |
| Case management | Produced health facility and home-based care tools that have been used in the most affected countries.  Conducted nine mpox clinical webinars, reaching over 3,000 attendees across 91 countries.  Updated the 2022 mpox case management treatment guidelines.  Developed and disseminated 6 clinical job aids to support frontline clinicians.    Delivered ToT to 50 clinicians across 12 Member States.  Trained 653 healthcare workers and 566 CHWs in DRC, while also supporting 824 clinicians in Liberia and 450 in Angola, Eritrea, Lesotho, and Uganda.  Assessed 168 health facilities in DRC using 5S methodology to identify gaps.  Delivered supplies to support 16,000 patients, nutritional support to over 2,000 patients and food vouchers to caregivers. | More than 3500 trained on mpox cases management with 50 health workers trained on integrated management.  Developed guidelines for optimization of standard supportive care for mpox.  Cascaded mpox integrated case management training in Liberia, Zambia, Tanzania, Sierra Leone with over 120 health care workers (HCW) trained.  Provided nutritional support 1500 mpox patients. |
| IPC | WHO IPC guidelines adopted in 16 of 19 targeted African Union Member States.  Conducted IPC facility assessments across African Union Member States using a standardized rapid mpox assessment tool in over 10 countries.  Defined a standardized list of essential IPC supplies for all affected countries to guide quantifications and delivery.  Developed a 5-day integrated training package incorporating all response pillars for both health workers and CHWs.  Trained 2,000+ frontline staff in IPC and conducted global webinars via WHO and Africa CDC platforms.  Distributed IPC/WASH supplies to CAR, Uganda, Burundi, and DRC.  Developed updated IPC tools, including home-care guidance and exposure definitions. | Developed infographic for PPE usage for Uganda.  built capacity for 150 persons in priority countries including Sierra Leone and Uganda.  Conducted IPC assessments in priority countries.  Deployment of IPC consultants in high burdened countries. |
